# Supplementary material for: Automatic semantic segmentation of breast tumors in ultrasound images based on combining fuzzy logic and deep learning—A feasibility study
Source: PLoS One. 2021 May 20;16(5):e0251899. doi: 10.1371/journal.pone.0251899 (PMC8136850; doi:10.1371/journal.pone.0251899)
Supplement: Attachment — Submitted filename: Response to Reviewers.pdf [file pone.0251899.s004.pdf]

Dear Editor (s),

Hoping you are all well;
Special thanks for your decision letter on our manuscript No. PONE-D-21-09994.

Concerning the following hint from you:
"We noted in your submission details that a portion of your manuscript may have been presented or published elsewhere. „Our submitted manuscript intitled: "Automatic Semantic Segmentation of Breast Tumors in Ultrasound Images Based on Combining Fuzzy Logic and Deep Learning – A Feasibility Study", is a replacement to the submission No. PONE-D-21-07670 (Efficient Automatic Semantic Segmentation of Breast Tumors in Ultrasound Images Based on Combining Fuzzy Logic and Deep Learning). „ Please clarify whether this publication was peer-reviewed and formally published. If this work was previously peer-reviewed and published, in the cover letter please provide the reason that this work does not constitute dual publication and should be included in the current manuscript."

We assure that this paper has not been presented or published elsewhere. The issue of the old withdrawn submission "PONE-D-21-07670" is that more changes have been needed. So, we have withdrawn it and modified it totally, and then we submitted it again to Plos One as: PONE-D-21-09994. So, again we assure that our submission has not been presented or published elsewhere.

Concerning Figures: All have been adapted by PACE (https://pacev2.apexcovantage.com/) – Special Thanks.

In the following tabulated sections, we will address most of the reviewers' comments.

Best Regards;
Corr. Author
Mohammed T. GadAllah

**Reviewer-1 Comments**	**Author Response**
1 - The abbrivation should be used only first time, then only acroyyms should be used in the entire paper.	**Thanks for your comment, kindly check all now.**
Abstract can be reduced. The abstract is NOT satisfactory because it didn't contain the following parts:<br>i. The importance of or motivation for the research.<br>ii. The issue/argument of the research.<br>iii. The methodology.<br>iv. The result/findings.<br>v. The implications of the result/findings.	**Thanks for your comment, kindly check the following modified abstract:**<br><br>**The first two sentence of the abstract can be considered the motivation and the issue:**<br>**<u>The motivation and the issue:</u>**<br>*Computer aided diagnosis (CAD) of biomedical images assists physicians for a fast facilitated tissue characterization. A scheme based on combining fuzzy logic (FL) and deep learning (DL) for automatic semantic segmentation (SS) of tumors in breast ultrasound (BUS) images is proposed.*<br>**<u>The methodology:</u>**<br>*The proposed scheme consists of two steps: the first is a FL based preprocessing, and the second is a Convolutional neural network (CNN) based SS. Eight well-known CNN based SS models have been utilized in the study. Studying the scheme was by a dataset of 400 cancerous BUS images and their corresponding 400 ground truth images. SS process has been applied in two modes: batch and one by one image processing. Three quantitative performance evaluation metrics have been utilized: global accuracy (GA), mean Jaccard Index (mean intersection over union (IoU)), and mean BF (Boundary F1) Score.*<br>**<u>The result/findings:</u>**<br>*In the batch processing mode: quantitative metrics' average results over the eight utilized CNNs based SS models over the 400 cancerous BUS images were: 95.45% GA instead of 86.08% without applying fuzzy preprocessing step, 78.70% mean IoU instead of 49.61%, and 68.08% mean BF score instead of 42.63%. Moreover, the resulted segmented images could show tumors' regions more accurate than with only CNN based SS. While, in one by one image processing mode: there has been no enhancement neither qualitatively nor quantitatively.*<br>**<u>Implications of the result/findings:</u>**<br>*So, only when a batch processing is needed, utilizing the proposed scheme may be helpful in enhancing automatic ss of tumors in BUS images. Otherwise applying the proposed approach on a one-by-one image mode will disrupt segmentation's efficiency. The proposed batch processing scheme may be generalized for an enhanced CNN based SS of a targeted region of interest (ROI) in any batch of digital images. A modified small dataset is available: www.kaggle.com/mohammedtgadallah/mt-small-dataset*

- In the first four paragraphs of literature review section, the authors have presented a good reference, **but they need to present the recent and most updated references**<br><br>■ Authors should add the most recent reference:<br>1) BCD-WERT: a novel approach for breast cancer detection using whale optimization based efficient features and extremely randomized tree algorithm, PeerJ Computer Science 7, e390<br><br>2) Classification of COVID-19 individuals using adaptive neuro-fuzzy inference system, Multimedia Systems, 1-15	Sir, special thanks for your comment. **But kindly be informed that the related work mentioned in the paper has about 12 researches (two of them 2021/ four of them 2020/ three of them 2019/and three 2018)**<br><br>**However, the two mentioned references (2021) by you have been added to reference list and cited in the paper as upon your demand (Ref. No. 40/41).**<br><br>**Also, Ref. No. 39 has been added 2021.**<br><br>*"R. Yang and Y. Yu, Artificial Convolutional Neural Network in Object Detection and Semantic Segmentation for Medical Imaging Analysis, Frontiers in Oncology, 11, Article 638182, 2021."*
- Authors should discuss the dataset extensively.	**Thanks for your comment. Kindly find the complete description for the images used in section III – under sub section 2 (the data set used)**
- The quality of the figures can be improved more. Figures should be eye-catching. It will enhance the interest of the reader.	**All are adapted by PACE (https://pacev2.apexcovantage.com/) – Special Thanks for Plos One**
Please highlight the contribution clearly in the introduction	***Kindly; revise this section in the introduction. More explanation can be founded in the methods section III***<br>*"In this paper, we introduce an automatic SS approach for batch processing by combining a Fuzzy method for contrast enhancement using an intensification operator as a preprocessing enhancement step before starting a known CNN based SS scheme. Eight CNN based SS schemes has been applied from [8]: FCN with AlexNet network, UNet network, SegNet using VGG16, SegNet using VGG19, DeepLabV3+ using ResNet18, DeepLabV3+ using ResNet50, DeepLabV3+ using MobileNet-V2, and DeepLabV3+ using Xception networks. The output segmentation results has been evaluated by three measures: global accuracy, mean IoU also called mean Jaccard Index, and mean BF (Boundary F1) Score. The proposed approach has been applied using a 400 BUS's images and their 400 ground truth images taken from [28] in two modes: batch processing and one by one image processing. A new dataset named MT_Small_Dataset (based on the 800 images taken from [28]) has been adjusted and arranged for 1200 images: 400 adjusted to size 128 by 128 by 3 and the same 400 (size 128 by 128 by 3) after applying fuzzy based contrast enhancement and 400 image ground truth adjusted in gray level [0 255], size (128 by 128), and have two classes "1" represents normal tissue and "2" represents cancerous tissue to be appropriate for evaluating the most known CNN based semantic segmentation output images using MATLAB.[29] "*

<table>
<tr><td>Some Paragraphs in the paper can be merged and some long paragraphs can be split into two.</td><td>Thanks for your comment. Kindly find the modified manuscript now.</td></tr>
<tr><td>The discussion is very important in research paper. Nevertheless, this section is short and should be presented completely.</td><td>Thanks for your comment. Kindly, be informed that the major of the information is concentrated into flowcharts and tables.</td></tr>
<tr><th>Reviewer-2 Comments</th><th>Author Response</th></tr>
<tr><td>In Introduction section, the drawbacks of each conventional technique should be described clearly.</td><td rowspan="2">Thanks for your two comments.

Kindly, be notified that: we here not concerned with comparison of CNN and any other technique. You can revise ref. no. 08 (W. Gomez-Flores and W Pereira): in their study they have compared between 8 CNNs models to obtain better SS.

Here, we applied the 8 models two times in two modes: i.e. four times. Resulted in: only when a batch processing is needed, utilizing the proposed scheme may be helpful in enhancing automatic ss of tumors in BUS images. Otherwise applying the proposed approach on a one-by-one image mode will disrupt segmentation’s efficiency. The proposed batch processing scheme may be generalized for an enhanced CNN based SS of a targeted region of interest (ROI) in any batch of digital images.</td></tr>
<tr><td>The authors should emphasize the difference between other methods to clarify the position of this work further.</td></tr>
</table>

The motivation for the present research would be clearer, by providing a more direct link between the importance of choosing your own method.	**Thanks for your comment, kindly check the modified abstract.**
Define all the variables before using	**Thanks for your comment. Kindly, find the modified manuscript now.**
The writing of the paper needs a lot of improvement in terms of grammar, spellings, and presentations. The paper needs careful English polishing since there are many typos and poorly written sentences.	**Thanks for your comment. Kindly, check the manuscript now.**
The authors can cite the following references<br><br>Antlion re-sampling based deep neural network model for classification of imbalanced multimodal stroke dataset<br><br>An AI-based intelligent system for healthcare analysis using Ridge-Adaline Stochastic Gradient Descent Classifier	**Thanks for your comment. Kindly, find the two mentioned references by you have been added to reference list and cited in the paper as upon your demand (Ref. No. 43/44).**
